# The Role of Thrombolytic Therapy for Patients with a Submassive Pulmonary Embolism

**DOI:** 10.7759/cureus.2814

**Published:** 2018-06-15

**Authors:** Elizabeth Murphy, Ahmed Lababidi, Renuka Reddy, Taaha Mendha, David Lebowitz

**Affiliations:** 1 University of Central Florida College of Medicine, University of Central Florida College of Medicine; 2 Office of Faculty and Academic Affairs, University of Central Florida College of Medicine, Orlando, USA

**Keywords:** pulmonary embolism, thrombolysis, heparin, tpa, mortality

## Abstract

A pulmonary embolism (PE) is an acute life-threatening respiratory event that results in upwards of 200,000 deaths per year in the United States. While anticoagulation is currently the standard of treatment for PEs, there is increasing evidence to suggest that in certain cases anticoagulation in combination with thrombolytic therapy may improve patient outcomes and reduce mortality. This article aims to compare the effects of combined intervention with thrombolytic therapy and anticoagulation to the effects of anticoagulation alone in patients with submassive PEs in terms of various outcome measures, including but not limited to: mortality, hemodynamic status, length of hospital stay, and safety. The methodology consisted of the critical appraisal of the primary literature articles pertaining to intervention with thrombolytic agents in cases of a submassive or intermediate risk PE, including a discussion of each study’s strengths and limitations. Ultimately, this review found that the use of thrombolytic agents in conjunction with anticoagulants has been associated with decreased hemodynamic decompensation and decreased length of hospital stay, with no change in mortality outcomes, at a cost of increased rate of bleeding and stroke. The use of thrombolytic agents with anticoagulants may be warranted in a specific subset of patients, but clinicians should consider the potential benefits and harms of this intervention.

## Introduction and background

In the United States, roughly 600,000 pulmonary embolisms (PEs) occur yearly and result in upwards of 200,000 deaths per year. Only a small fraction of PEs, roughly 25%, are recognized and diagnosed, while the majority of cases are identified at autopsy [1]. PEs fall under the broader category of venous thromboembolisms, which also include deep vein thromboses. Most PEs are secondary to thrombus development in the deep venous system in the lower extremities and the pelvis, with a small proportion (3-33%) arising from thrombi originating from the upper extremities. From the extremities, the thrombi embolize through the inferior vena cava and right heart, resulting in pulmonary arterial obstruction, which is by definition, a PE [2]. Hereditary risk factors for PEs include antithrombin deficiency, protein C or S deficiency, factor V Leiden, and prothrombin gene mutation. Acquired risk factors for PEs include age, obesity, pregnancy, immobilization, surgery, malignancy, oral contraceptives, hormone replacement therapy, and central venous catheters [1].

PEs can be divided into massive, submassive, and low risk. A massive PE is defined as an acute PE with sustained hypotension with a systolic blood pressure <90 mm Hg for at least 15 minutes or requiring ionotropic support [3]. Patients with submassive PE have normal blood pressure and evidence of right ventricle dysfunction, which may be evidenced by electrocardiogram, computed tomographic pulmonary angiography transthoracic echocardiogram and transesophageal echocardiogram. Elevated cardiac biomarkers, such as brain natriuretic peptide and troponin I, can also suggest a submassive PE and have been correlated with increased mortality. Submassive or intermediate-risk PEs comprise 31% of diagnosed PE cases and have a 5% to 12.6% in-hospital mortality rate [1]. A low-risk PE can be defined as an acute PE in the absence of clinical markers that define massive or submissive PE, as mentioned above [3].

The clinical presentation of a PE can vary widely between cases. A PE is typically acute in onset and common symptoms include dyspnea, pleuritic chest pain, palpitations, cough, wheezing, and orthopnea. Vital sign abnormalities can include tachycardia, tachypnea, hypotension, and hypoxemia. Massive PEs can present with mental status changes, syncope, arrhythmias, or death [1]. The initial presentation of a PE is sudden death in roughly 25% of cases [2]. Other features of the physical examination that may suggest a PE include accessory muscle use, decreased breath sounds, and a right ventricular heave. The diagnosis of a lower extremity deep vein thrombosis (DVT) can put patients at an increased risk for developing a PE; a DVT may present with asymmetric calf swelling or pain. The clinical presentation of a PE can be similar to that of other life-threatening conditions, such as an aortic dissection, myocardial infarction, or pericardial tamponade [1].

Clinical signs and symptoms can raise suspicion for a PE, but the diagnosis must be ultimately confirmed via other modalities. Clinical decision tools, such as the Wells Score, PERC score, and the Revised Geneva Score, can aid physicians in determining a patient’s risk for having a PE [1]. The PERC score has been widely adopted by emergency physicians and takes into consideration factors such as age, recent history of trauma, oral contraceptive use, prior DVT, vital signs, and the presence of hemoptysis. Another tool to aiding the diagnosis of PE is the quantitative enzyme-linked immunosorbent assay test for D-dimer, a laboratory test with high sensitivity (95%) but low specificity for a PE. The test has a low specificity because elevated D-dimer is also commonly observed in trauma patients, post-surgical patients, critically ill patients with malignancy, infection, and an acute myocardial infarction. Elevated D-dimer is also a common finding in venous thromboembolism and in pregnancy [1]. In the past, ventilation-perfusion lung scanning was used to diagnose PE with a high-probability scan being strongly correlated with a diagnosis of PE and a normal scan eliminating the diagnosis of PE. Since 50% of examinations are non-diagnostic and thus cannot by themselves exclude or diagnose PE, ventilation-perfusion scans have slowly fallen out of favor amongst clinicians [4]. In recent years, imaging studies have become the standard for diagnosing a PE and include pulmonary angiograms and computed tomographic pulmonary angiography [1].

According to the American Heart Association, the current recommendation, established in 2011, once a PE has been diagnosed and the patient does not have any contraindications, is to administer anticoagulant therapy with subcutaneous low-molecular-weight heparin (LMWH), intravenous or subcutaneous unfractionated heparin (UFH) with monitoring, unmonitored weight-based subcutaneous UFH, or subcutaneous fondaparinux [3]. Heparin is an anticoagulant that functions by inactivating thrombin and activated factor X through an antithrombin-dependent mechanism [5]. In some cases, clinicians choose to administer a fibrinolytic agent in addition to heparin anticoagulation, but this is not the current standard treatment in all PE cases. Fibrinolytic agents are enzymes that convert a patient’s native circulating plasminogen into plasmin and are comprised of three major classes of drugs, tissue plasminogen activators, streptokinase, and urokinase. Tissue plasminogen activators include drugs such as alteplase, reteplase, and tenecteplase. While heparin causes a passive reduction of thrombus size, thrombolytic agents promote the hydrolysis of fibrin molecules [3].

The optimal use of thrombolytic agents remains uncertain and the potential benefits and harms must be considered prior to use of thrombolytic agents. Potential benefits to using thrombolytics include more rapid symptom resolution, stabilization of cardiac and respiratory function without the need for external support, improved exercise tolerance, and increased survival probability. The main potential harm of thrombolytic therapy is the risk of hemorrhage, both fatal and minor, and the subsequent prolongation of hospitalization. There is some evidence to suggest that patients treated with thrombolysis may have a decrease in all-cause related mortality, especially in massive PE [3]. The American Heart Association states that fibrinolysis is reasonable for patients with massive acute PEs and may be considered for patients with submassive acute PEs with clinical evidence of adverse prognosis in patients with a low to acceptable risk of bleeding complications [3]. Thus, critical analysis of the role of intervention with thrombolytic agents in PE cases is warranted.

## Review

This critical appraisal of the literature aims to explore whether using thrombolytic agents in conjunction with anticoagulants is more effective than anticoagulant therapy alone for reducing mortality in hemodynamically stable patients with acute submassive PEs. Our review focuses on comparing various outcomes including, but not limited to, mortality, hemodynamic status, length of hospital stays, and safety with the use of a combination of heparin and thrombolytics vs. heparin alone in the setting of an acute PE.

A 2002 study conducted by Konstantinides et al., studied the use of heparin combined with thrombolytics compared to heparin alone on the effects on mortality in patients with submassive PEs. This prospective, double-blinded 49 center study randomly assigned patients to the treatment group (118 patients) receiving heparin plus 100 mg of alteplase, and the control group (138) receiving heparin plus a placebo. The inclusion criteria included patients with an acute PE plus one of the following: right ventricular dysfunction on echocardiogram, pulmonary-artery hypertension on echocardiogram or catheterization, or electrocardiographic signs of right ventricular strain. There was no loss to follow-up. All patients were started on an initial bolus of 5000 U of heparin then assigned to either the treatment or the placebo group. The treatment group received a 10 mg bolus of alteplase followed by a 90 mg infusion over a period of two hours. The placebo group received the same volume of a placebo. Additionally, both groups were continued on a heparin drip and an oral anticoagulant was started in both groups three days following randomization. Patients were either evaluated at the end of their hospital stay or after 30 days depending on which occurred first. There were no significant differences between the two arms in baseline characteristics [6].

The primary outcome of this study was in-hospital death and clinical deterioration requiring further treatment. Rates of major bleeding and PE recurrence measured as secondary end points. Overall the incidence of the primary end point (death or escalation of treatment) was significantly greater in the heparin-plus-placebo group than in the heparin-plus-alteplase group (p = 0.006). However, looking at death alone as an end point, this study failed to show a significant difference (p = 0.71) as four patients from the treatment and three patients from the control group died. Although the mortality rate was similar between treatment groups, the rate of escalation of treatment because of clinical deterioration was much higher in the placebo group at 24.6% compared to the treatment group at 10.2% with a number needed to treat (NNT) of 7 (P = 0.004). Importantly, this double-blinded study became open label, once there was an escalation of treatment, leading to possible bias. The probability of a 30-day event-free survival according to the Kaplan-Meier analysis was significantly higher in the heparin plus alteplase group than in the heparin plus placebo group (p = .005). There was no significant difference in the occurrence of any of the secondary criteria including recurrent PEs, minor, and major bleeding between the two groups [6].

Overall, this study demonstrates that while the use of thrombolytics combined with heparin is not superior over heparin alone in reducing mortality following a submassive PE, thrombolytics plus heparin are superior in preventing the need for escalation of treatment including the need for secondary thrombolysis. Additionally, the use of heparin with thrombolytics does not have a substantial increased risk of bleeding compared to heparin alone. Based on these findings, there is evidence that the use of thrombolytics can be recommended in the setting of an acute submassive PE in a patient with no contraindications.

In 2013, Sharifi et al. published “Moderate Pulmonary Embolism Treated With Thrombolysis" (MOPETT trial). This is a single center, randomized control trial, that enrolled 121 adult patients with moderate PE defined as signs and symptoms of PE plus computed tomography pulmonary angiographic involvement affecting > 70%, involvement of thrombus in greater than two lobar or left or right main pulmonary arteries or by a high probability V/Q scan showing a V/Q mismatch in greater than two lobes. The study questioned whether a low dose of tissue plasminogen activator 0.5 mg/kg (max 50 mg), given as a 10 mg bolus, followed by a drip over two hours, would lead to differences in rates of pulmonary hypertension, mortality, and bleeding. An echocardiogram was done before administration of tissue plasminogen activator, repeated during hospital stay, and then repeated at six-month intervals, with a length of follow-up of around 28 months. Both arms of the trial received warfarin plus either heparin or enoxaparin. The baseline characteristics between the two arms were statistically similar [7].

Pulmonary hypertension occurred in 16% and 57%, NNT of two (p < 0.001) in the tissue plasminogen activator arm and control arm, respectively. Hospital length of stay was 2.2 versus 4.9 days (p < 0.001) in the tissue plasminogen activator arm and control arm, respectively. There was no significant difference when looking at recurrent PE and all-cause mortality alone, however, when studying both of them together as a composite outcome, there was a statistically significant difference of 1.6% versus 10% (p = 0.049), in the tissue plasminogen activator arm and control arm, respectively. Importantly, there was no major bleeding or intracranial hemorrhage in both groups. This study was one of the first studies to examine the use of a “safe dose” tissue plasminogen activator, which is 50% less than the traditional dose. Although the study revealed some benefit when looking at pulmonary hypertension and hospital length of stay, there were several limitations. First, pulmonary hypertension is not a patient-oriented outcome, as it is difficult to determine how this actually affects the patients and if this causes any symptoms or long-term consequences for them. Additionally, this study was unblinded and performed at a single center with a small sample, limiting its power and external validity. Unlike other similar studies, their definition of moderate PEs did not include those patients with right ventricular strain or elevated troponin levels, making it hard to compare to other studies utilizing thrombolytics [7].

Meyer et al. published a study in 2014 titled, “Fibrinolysis for Patients with Intermediate-Risk Pulmonary Embolism”, also referred to as the International PEITHO (Pulmonary Embolism Thrombolysis) trial aimed to explore the role of fibrinolytic therapy in patients with intermediate-risk pulmonary embolism. This study is a double-blinded trial, which compared tenecteplase plus heparin with placebo plus heparin in normotensive patients with intermediate-risk PEs. Eligible patients had right ventricular dysfunction on echocardiography or computed tomography, as well as myocardial injury as indicated by a positive test for cardiac troponin I or troponin T. The primary outcome was death or hemodynamic decompensation (or collapse) within seven days after randomization. The main safety outcomes were major extracranial bleeding and ischemic or hemorrhagic stroke within seven days after randomization. The sample size of this study was 1005 patients [8].

Hemodynamic decompensation occurred in 1.6% in the tenecteplase group as compared with 5.0% in the placebo group (p = 0.002), however looking at death alone in seven and 30 days there were no significant differences between the two groups. Extracranial bleeding occurred in 6.3% in the tenecteplase group and 1.2% in the placebo group (p < 0.001). Stroke occurred in 2.4% in the tenecteplase group and in 0.2% in the placebo group (p = 0.003). In summary, although there was a reduction in hemodynamic decompensation, there was no mortality benefit. In fact, tenecteplase was associated with a significant increase in the risk of intracranial and other major bleeding. This study has many strengths. The sample size of 1005 is considerable, compared to other published studies in this field. Splitting the sample size into age subgroups provided significant data that allowed the authors to conclude that the risks of complications from thrombolytic therapy are less in the younger population compared to the older one. A limitation to the study was that the patients were very closely monitored. The authors discuss the possibility of some patients having worse prognosis had they not been closely monitored and promptly treated when decompensation occurred. Though an unavoidable practice, this exceptional increase in attention to patients of this study might confound the data. Another limitation of this study is the monitoring of patients until 30 days post PE, making the mid-to-long term outcomes of thrombolytic therapy versus anti-coagulation unexplored [8].

To address long-term outcomes of systemic thrombolysis in intermediate-risk PE patients, a study by Konstantinides et al., in 2017, followed 709 of 1005 patients from the PEITHO trial above, for a two-year follow-up period. Again, this was a double-blind randomized controlled trial looking at adult patients with confirmed PE with right ventricular dysfunction on echocardiogram and/or computed tomography scan along with myocardial injury as evidenced by an elevated troponin level. Survival up to a median of 37.8 months was studied and revealed a mortality rate of 20.3% and 18% in the thrombolysis and placebo arm, respectively (p = 0.43). The long-term clinical status was obtained in 175 and 183 patients in the tenecteplase and placebo arms of the trial, respectably, with 36% of them in the tenecteplase arm and 30.1% in the placebo arm reporting persistent symptoms, such as dyspnea, however, this was not statistically significant. Echocardiogram was also performed on a subset of these patients to determine if there were any differences in pulmonary hypertension or right ventricular dysfunction, with again, no differences between the two arms of the trial. In summary, this long-term follow-up to the original PEITHO trial did not reveal any statistically significant differences between the two arms of the study. The study was limited as there was a loss to follow-up especially when looking at the clinical status and echocardiogram results, putting into question the validity of this portion of the results. Although there are concerns with validity, this study is one of the only studies that look into patient outcomes that matter other than mortality, such as persistent dyspnea [9].

Furthermore, in 2014 a study entitled “Treatment of submassive pulmonary embolism with tenecteplase or placebo: cardiopulmonary outcomes at three months: multicenter double-blind, placebo-controlled randomized trial” (TOPCOAT) was published with an objective to evaluate whether tenecteplase increases the probability of a favorable composite patient-oriented outcome after submassive PE. This study is a double-blinded, placebo-controlled randomized trial resulting from the participation of eight centers. This study enrolled 83 normotensive patients with a PE and right ventricular strain, confirmed by echocardiography, elevated troponin or elevated brain natriuretic peptide levels. All patients received low molecular weight heparin followed by random assignment to either a single weight-based bolus of tenecteplase or placebo, administered in a double-blinded fashion. The primary composite outcome included: death, circulatory shock, intubation or major bleeding within five days or recurrent PE, poor functional capacity (right ventricular dysfunction with either dyspnea at rest or exercise intolerance) or a Short Form 36 (SF-36) health survey Physical Component Summary (PCS) score of < 30 at 90-day follow-up. From the 83 patients randomized, 40 received tenecteplase and the rest received placebo. There were no significant baseline characteristic differences between the patients except those in the tenecteplase arm had a higher rate of malignancy under chemotherapy treatment at 12.5% compared to zero in the control arm (p = 0.01) [10].

Results showed that 37% of the patient treated with placebo and 15% treated with tenecteplase had an adverse composite outcome within five days and at 90-day follow-up (p = 0.017). Within five days of the trial, adverse outcomes occurred in three placebo-treated patients (death in one and intubation in two) and one tenecteplase-treated patient (fatal intracranial hemorrhage). At 90-day follow-up there were no clinically important differences between the two arms in the mean six-minute walk distance or the score on the SF-36 Mental Component Summary, however there was a modest difference on the overall health self-assessment survey which was scored from 1-10, with the placebo group achieving a score of 2.4 and the tenecteplase group with a score of 3.3 (p = 0.036) [10].

This study attempted to elucidate long-term prognosis in patients receiving thrombolytics for submassive PEs. There were modest improvements on a self-assessment survey and less adverse events at five and 90 days in those receiving tenecteplase. There was one patient in the tenecteplase arm who died from intracranial hemorrhage, but this was balanced by a death in the placebo group, as well as two patients in the placebo group requiring intubation. This study is limited by a small sample size, decreasing its power. The study was prematurely terminated due to the principal investigator relocating to a new institution [10].

A 2016 study conducted by Sinha et al., studied the impact of thrombolytic therapy combined with heparin versus heparin alone in the treatment of acute, submassive PEs. This prospective, randomized, single-blinded study included 86 adult patients with PEs and evidence of right ventricular dysfunction. All participants were allocated either into the treatment group (heparin with tenecteplase) or the control group (heparin alone) within one hour of diagnosis. The two groups were matched to have similar average age, gender ratio, systolic blood pressure (SBP), heart rate, and risk factors. The primary outcome of this study was mortality from any cause of death, hemodynamic compromise or collapse (defined by need for cardiopulmonary resuscitation (CPR), persistent SBP < 90 mm Hg, or signs of shock) within seven days of enrollment in the study. Important secondary outcomes included recurrent PE within seven days, mean hospital stay, improvement in right ventricular function and death within 30 days. Both groups received the same dose of unfractionated heparin. The treatment group then received a weight-adjusted bolus dose of tenecteplase. The control group received the same volume of placebo (normal saline), followed by a maintenance dose of heparin [11].

Overall, there were 45 patients in the tenecteplase arm and 41 patients in the placebo arm. There was no significant difference in the primary outcome, as both arms experienced two deaths. There was a significant difference (p = 0.004) in the number of patients that experienced hemodynamic decompensation with two patients in the tenecteplase arm and eight patients in the placebo arm. Thus, tenecteplase and heparin reduced the risk of hemodynamic compromise over just heparin alone by 77.5%. There was also a significant difference between mean hospital stay in the tenecteplase group at 8.1 days versus the placebo group at 11.1 days (p = 0.001). Lastly, there was a statistically significant improvement in right ventricular function between both groups (p = .001) with 70% from the tenecteplase arm experiencing improvement in right ventricular function while 40% experienced an improvement in the placebo arm (p = 0.001) [11].

Although there was no statistically significant difference in mortality amongst the two groups, the tenecteplase group did experience a significant decrease in hemodynamic decompensation, length of hospitalization, and improvement on right ventricular function. Importantly, there was no statistically significant difference in the incidence of major bleeding between the groups in this study [6]. In summary, this study showed that there was a benefit to using a combination of tenecteplase with heparin as opposed to heparin alone in submassive pulmonary embolisms in order to improve right ventricular function, decrease length of hospital stay, and decrease rates of hemodynamic decompensation. However, there was no major benefit in tenecteplase with heparin in reducing overall mortality. The major strengths of this study include the stratification used to create equal groups, however, the study was limited by a very small sample size in a single center.

## Conclusions

This review aimed to assess the different outcomes associated with the use of thrombolytic therapy for the treatment of a submassive pulmonary embolism. More specifically, this review sought to analyze the risks versus benefits that are associated with each treatment regimen. Multiple studies have found no statistically significant reduction in mortality rates associated with thrombolytic therapy. However, those patients treated with thrombolytic therapy did have statistically significant decreased hemodynamic decompression and decreased length of hospital stay. Those treated with thrombolytics also had statistically significant improved right ventricular function and decreased rates of pulmonary hypertension. Importantly, thrombolytics have been associated with increased rates of bleeding, however, the key to avoiding this outcome may be from using a lower dose of thrombolytics as mentioned in the MOPPET trial, which showed no major bleeding. Therefore, at this moment it is difficult to draw definitive conclusions, except to recommend further trials be performed specifically looking at half or lower dose thrombolytics. Additionally, further trials comparing outcomes between systemic thrombolysis and newer ultrasound assisted catheter-directed thrombolysis in the treatment of pulmonary embolisms should be explored in order to draw further conclusions.
